# Effect of stereotactic radiotherapy on immune microenvironment of lung cancer

**DOI:** 10.3389/fimmu.2022.1025872

**Published:** 2022-09-23

**Authors:** Yao Xiao, Hongqing Zhuang

**Affiliations:** Department of Radiation Oncology, Peking University Third Hospital, Beijing, China

**Keywords:** immune microenvironment, lung cancer, stereotactic radiotherapy, SBRT, hypofractionated radiotherapy (HFRT), immunotherapy

## Abstract

Stereotactic radiotherapy (SRT) is one of the main treatment modalities for lung cancer, and the current SRT approach combined with immunotherapy has initially presented good clinical efficacy in lung cancer. SRT activates the immune system through *in situ* immunization, releasing antigens into the blood, which promotes the antigen–antibody response and then induces tumor cell apoptosis. Dose fractionation has different effects on the immune microenvironment, and the tumor microenvironment after SRT also changes over time, all of which have an impact on SRT combined immunotherapy. Although much research on the immune microenvironment of SRT has been conducted, many problems still require further exploration.

## Introduction

Stereotactic radiotherapy (SRT) is one of the main methods used to treat lung cancer, and SRT combined with immunotherapy has presented good clinical efficacy in the treatment of lung cancer (1–4). Compared to conventional radiotherapy, SRT is characterized by a large single dose and a small number of fractionations. Traditionally, radiotherapy has achieved local tumor control by inducing irreversible DNA damage in irradiated tumor cells. However, the radiobiological mechanism of SRT has not been fully elucidated. Recent studies have reported that SRT changes the tumor microenvironment (TME) (5–7), activating the immune system *via in situ* immunization and releasing antigens into the blood, promoting antigen–antibody responses, and inducing apoptosis of tumor cells through anti-tumor immunity (8) and vascular injury (9). Dose fractionation of SRT results in different effects on the immune microenvironment (10), and the TME after SRT also changes over time, both of which have an impact on the effect of SRT combined with immunotherapy. Although there have been many studies on SRT in the immune microenvironment, many problems require further exploration. Here, we review the literature and summarize the research progress of SRT combined with immunotherapy to improve the understanding of the SRT effect on the immune microenvironment of lung cancer.

## Effect of SRT on immune microenvironment of lung cancer

The TME is the internal environment for tumor survival and progression, and is related to tumor growth and metastasis (11). The tumor immune microenvironment (TIME) in the TME is composed of a series of immune cell types that have various roles. Effector cells with cell-killing functions kill cancer cells through different mechanisms in both innate and adaptive immune responses (12). Immunosuppressive cell populations in the TIME include CD4+ FOXP3+ regulatory T cells (Tregs), myeloid-derived suppressor cells (MDSCs), anti-inflammatory macrophages, and some B-cell subsets (12), as well as antigen-presenting cells such as dendritic cells (DCs), which play an important role in maintaining adaptive immune responses in the TIME (12). Moreover, the radiosensitivity of these cells is significantly different. Generally, NK and B lymphocytes are the most radiosensitive immune cells and DCs and Tregs are more radioresistant (13). Thus, the changes in the TIME after radiotherapy vary depending on dose fractionation, because dose fractionation leads to different proportions of various cell types in the TIME.

### Effect of SRT on *in situ* immune microenvironment

The main mechanism of radiotherapy is to induce irreversible DNA damage in tumor cells directly or indirectly through free radicals. SRT induces immunogenic cell death and activates the adaptive immune response by promoting cross-presentation of tumor antigens by DCs to T cells (14). The antitumor response of effector T cells includes recognition of tumor antigens and attacking of cancer cells, which is related to MHC-I, the PD-1/PD-L1 axis, and T cell receptor (TCR). Elevated MHC-I expression in tumor tissues after stereotactic body radiotherapy (SBRT) promotes the recognition of CD8+ T cells by *in situ* tumor-specific antigens. In early preclinical studies, the effects of hypofractionated radiotherapy protocols on the TIME are better than those of the conventional protocol, and the expression of MHC-I and related tumor peptides is higher with increasing radiotherapy dose (15, 16). A higher dose has been reported to enhance the upregulation of other immune signals, thereby improving tumor-specific CD8+ T cell infiltration (17, 18). SRT improves TCR sequence diversity and PD-L1 expression in the TME of lung cancer patients (5), but its promotion of the infiltration of CD8+ T cells and NK cells in the TME has not been observed, possibly because of differences in fractionated radiotherapy doses or sampling time points. A recent phase II randomized trial of SBRT combined with durvalumab or durvalumab alone in patients with early-stage operable NSCLC reported major pathological response rates of 53.3% (95%CI 34.3%–71.7%) and 6.7% (95%CI 0.8%–22.1%), respectively, with statistical significance (19). Importantly, significantly higher MHC-I gene expression was identified in post-treatment tumor specimens of patients with significant pathological remission in the combination group. Furthermore, CD8+ T cells recognized tumor neoantigens that were upregulated by RT when metastatic NSCLC patients were treated with the same treatment (20).

### Effect of SRT on humoral immunity

**Figure 1:**
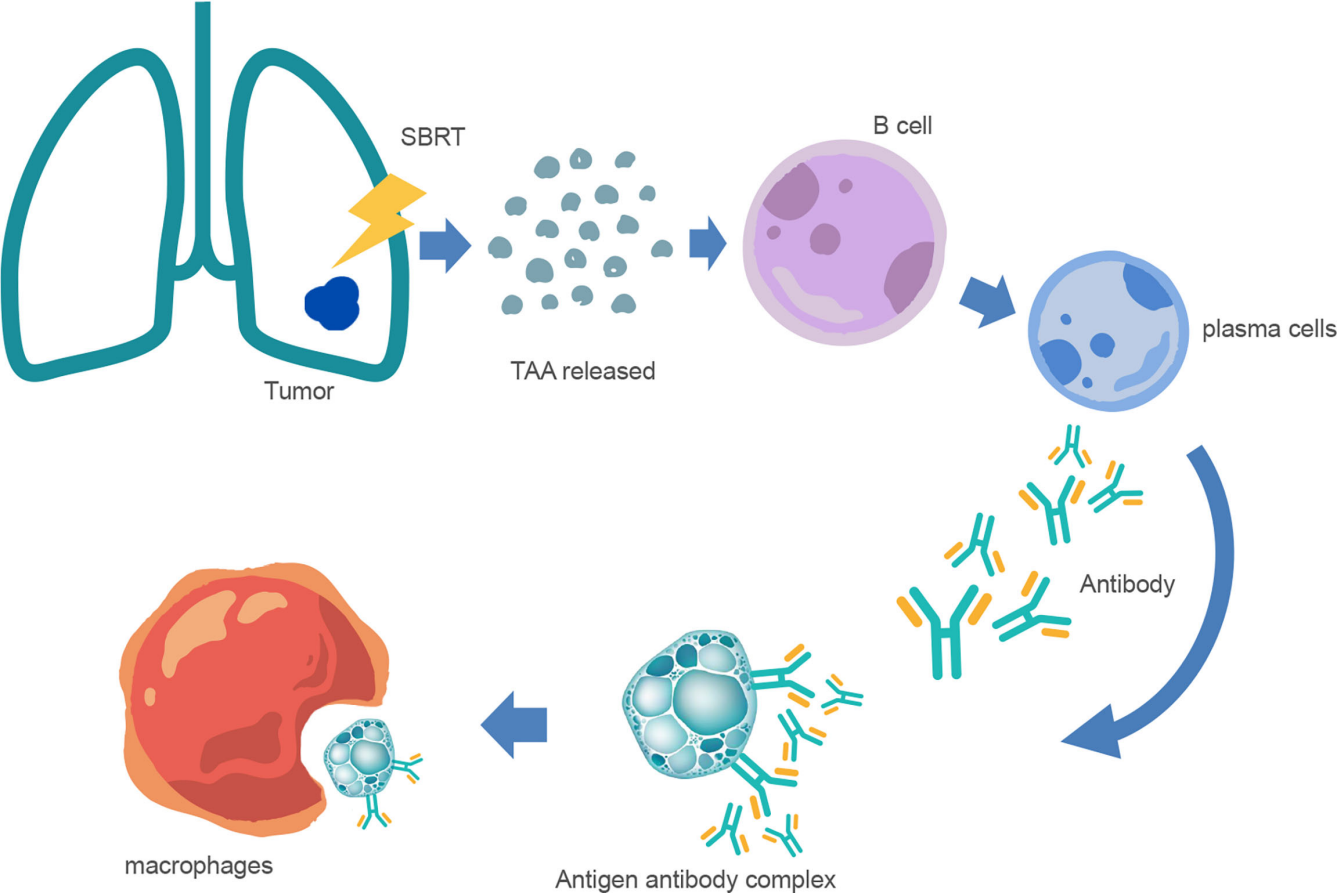
Mechanism of anti-tumor humoral immunity after SBRT. After SBRT, many tumor-associated antigen (TAA) fragments are released into the blood and stimulate the differentiation of B lymphocytes into plasma cells. Furthermore, plasma cells produce antibodies and bind to tumor cells to form antigen–antibody complexes, and are then phagocytosed and cleared by macrophages.

Increasing evidence indicates that SRT can induce significant antitumor effects (5, 16, 21, 22). Generally, cellular immunity is dominant among antitumor immunity, and humoral immunity usually plays a synergistic role only in some cases. For most tumors with strong immunogenicity, specific immune responses are predominant, but for those with weak immunogenicity, non-specific immune responses may be of higher importance. Humoral immunity is a non-specific immune response, and circulating antibodies mainly produce immune responses against tumor cells with weak antigenicity in a free state (**Figure 1**). B lymphocytes are not only antigen-presenting cells but also important antibody-producing cells. Studies have indicated that tumor immunity is induced in NSCLC patients receiving SBRT through upregulated IgG and/or IgM (23). Zhang et al. (24) analyzed the peripheral blood immune cells of NSCLC patients after hypofractionated SRT (HSRT) and found that the proportion of naive B cells and double-negative B cells was low after moderate-dose HSRT (48 Gy/8 F or 48 Gy/6 F). However, the proportions of MZ-like B cells, transition B cells, and plasmablastic cells were higher. Lei et al. (25) found that compared with single low-dose irradiation (2 Gy), single high-dose irradiation (10 Gy) significantly stimulated the secretion of A549-related exosomes in a dose rate-dependent manner. Exosomes derived from ultra-high dose-rate radiation contribute to the polarization of B and NK cell subsets in peripheral blood mononuclear cells, thereby achieving greater antitumor immune responses.

## Differences in SRT dose effects on the immune microenvironment

Radiotherapy in clinical treatment aims to maximize the damage to the target area and minimize the damage to the adjacent healthy tissue. Generally, stereotactic radiosurgery (SRS) or stereotactic ablative radiotherapy refers to a one-time dose of 20–34 Gy or a single dose of 10–15 Gy for a total of 3–5 times (26, 27), as well as hypofractionated protocols, i.e., a single dose of >2 Gy and <10 Gy, accumulating until the total dose of ablation (defined as the dose achieving >90% local control) or the total sub-ablation is achieved depending on the tumor location, type, and therapeutic purpose. These radiotherapy protocols differ in their immunomodulatory effects (28) (**Table 1**). Most preclinical studies of the immunomodulatory effects of SRT applied solid tumor models and single RT doses of 10 to 20 Gy or hypofractionated non-ablative protocols (e.g., 8 Gy × 3 F). Among them, the 8 Gy × 3 F hypofractionated radiotherapy protocol is a common immunomodulatory protocol in early preclinical studies (36) that can induce a stronger IFN-I response than the conventional protocol (37). Ablation doses, such as 20 Gy/Fx, lead to severe cell death and depletion of radiation-resistant inhibitory immune cells in the TME, but may also result in elevated levels of fibrosis and chronic inflammatory/immunosuppressive pathways (38, 39). In 2020, Lu et al. initiated a triple therapy of low-dose radiotherapy, hypofractionated radiotherapy and immunotherapy, and preliminarily verified its efficacy in a tumor-bearing mouse model and retrospective clinical data (10). This study revealed the potential mechanism of therapy combining different radiotherapies and immunotherapy: hypofractionated radiotherapy induces apoptosis of *in situ* tumor cells, exposes tumor-specific antigens, produces an “*in situ* vaccination” effect, and sensitizes tumor-specific T cells; low-dose radiotherapy promotes migration of tumor-specific T cells into the distal tumor and regulates the immune microenvironment of the distal tumor, which jointly produces CD8+ T cell-dependent immune effects; and PD-1 inhibitors restore the tumor-killing activity of T cells by loosening the “inhibitory brake” on the surface of T cells and further enhancing systemic anti-tumor effects. The results of this study suggest the potentially important clinical application value of low-dose radiotherapy in immune activation. Thus, high- or low-dose treatment is ultimately determined by the purpose of clinical treatment.

**Table 1:** Pre-clinical and clinical reports using RT ± immunotherapy as radiation dose difference of immune activation.

Author	Year	Type	RT	ICI	Outcome
Zhang et al. (24)	2017	Clinical	6 Gy × 8 f or 8 Gy × 6 f	None	Increased the frequency of CD8+ T cells, but decreased the frequency of inhibitory Tregs. Increased the proportions of MZ-like B cells, transitional B cells and plasmablast cells. Activated the peripheral immune response.
Formenti et al. (20)	2018	Clinical	6 Gy × 5 f or 9.5 Gy ×3 f	CTLA-4	Induced systemic anti-tumor T cells
Iyengar et al. (29)	2018	Clinical	SAbR: 18–24 Gy/1 f, 24.6–33 Gy/3 f, 30–37.5 Gy/5 f. HSRT: 45 Gy/15 f	None	Perlonged PFS
Navarro-Martín et al. (21)	2018	Clinical	7.5 Gy × 8 f or 12.5 Gy × 4 f	None	Increased the immune active components of the immune system,and decreased the Tregs, granulocytic myeloid-derived suppressor cells (G-MDSCs) and monocytic (Mo-MDSCs).
Wang et al. (30)	2019	Pre-clinical	8 Gy × 3f	PD-1	Increased lung injury score
Bauml et al. (31)	2019	Clinical	Stereotactic or standard fraction, dose NS	PD-1	Perlonged PFS
Theelan et al. (32)	2019	Clinical	8 Gy × 3 f	PD-1	No significant ORR improvement.
Lockney et al. (23)	2019	Clinical	6-12Gy / f	None	Induced tumor immunity through upregulated IgG and/or IgM.
Savage et al. (33)	2020	Pre-clinical	22 Gy × 1 f and 0.5 Gy × 4 f	None	Significant tumor growth delay and increased survival. Increased infiltration of immune effector cells and decreased Tregs in irradiated tumors and secondary lymphoid organs.
Zhou et al. (5)	2021	Clinical	6 Gy × 10 f	None	Improved TCR sequence diversity and PD-L1 expression in TME.
Schoenfeld et al. (34)	2022	Clinical	0.5 Gy × 4 f 8 Gy × 3 f	PD-L1 and CTLA-4	No significant RT toxicity. No benefit of adding RT.
Zhao et al. (35)	2022	Clinical and pre-clinical	mouse model: 3.7 Gy× 4 f, 4.6 Gy × 3f, 6.2 Gy×2f, and 10 Gy × 1f Patient: 3.7 Gy × 8f, 4.6 Gy × 6 f, 6.2 Gy × 4 f, and 10 Gy × 2 f	None	Induced the increase in CD8+ T cells and positive immune cytokine response.

## Changes in immune microenvironment correlated with different phases of SRT

The TIME has different manifestations before and after SRT. In a preliminary study, changes in circulating blood immune cell populations were observed in lung tumor patients treated with SRT, which included an increase in immunoreactive components and a decrease in immunosuppressive components. Although these changes did not show statistical significance, they appeared 72 h after SBRT and continued until 6 months after treatment (21). Zhang et al. (24) analyzed peripheral blood immune cells from six NSCLC patients with stage I disease who received HSRT and found that HSRT greatly activated the immune response 3 weeks after treatment. Another preclinical study (33) reported increases in cell-surface markers of immune regulation (CD80), stress (CRT, HSP70, FAS, and MHC-I), and immunosuppression (CD47 and PD-L1) at 6 h after radiotherapy in 3LL tumor-bearing mice treated with ablative radiotherapy. Moreover, mice sequentially exposed to low-dose post-ablation modulation had significantly delayed tumor growth and improved survival. Moreover, by increasing the infiltration of immune effector cells and reducing Tregs in irradiated tumors and secondary lymphoid organs, TME remodeling was promoted, and immunogenic potential was improved after ablative radiotherapy even 6 days after the initiation of radiotherapy (33). A recent study (35) analyzed the dynamic changes in the TIME after hypofractionated radiotherapy in mice and NSCLC patients. The results showed that HFRT induced an increase in CD8 T cells and positive immune cytokine responses at specific periods and fractionated doses. In this study, the optimal time window of the immune response was from 48 hours to 2 weeks, especially in the 6.2 Gy group. The optimal immune response was observed in the 10 Gy × 2 group after 96 h, and the intervention with immunotherapy may achieve better outcomes within such a time window. These studies provide a reference for the timing of SBRT combined with immunotherapy in the clinic.

Buchwal et al. reviewed several preclinical studies and suggested that, ideally, anti-PD-1/L1 and RT should be conducted simultaneously; if not, at least RT should be conducted at first (40). In a retrospective analysis of 125 patients who received SBRT/SRS immunotherapy including 90 patients (72%) with lung cancer, patients who completed immunotherapy before SBRT/SRS appeared to have worse OS than those receiving immunotherapy simultaneously or later (41). Multivariate analysis indicated that the timing of immunotherapy is still a significant prognostic factor for OS when considering age, sex, cancer type (lung cancer and other cancers), and radiotherapy type (P = 0.0), which is consistent with previous preclinical studies. Given that immunomodulators have different targets, the sequence of immunotherapy and radiotherapy depends on the mechanism of immunomodulators.

## Discussion

With the emerging efficacy of SRT combined with immunotherapy, the mechanism of SRT effects on the immune microenvironment needs to be explored. Although there have been many studies on the SRT immune microenvironment, there are still some problems.

### Insufficient research on the SRT immune microenvironment

Although there are increasing preclinical and clinical data on the combination of radiotherapy and immunomodulators, and the application prospect is exciting, most of the available data are currently from retrospective or small cohorts. Many problems related to the use of SRT to improve clinical conversion to immunotherapy efficacy remain unsolved, such as dose fractionation, treatment sequence, selection of immunomodulators, and biomarkers predicting treatment response (42). In particular, there are few studies about the immune microenvironment of SRT in some special sites of metastases. For example, brain metastases (BM), as the brain is a common distant metastatic organ of NSCLC. Compared with extracranial tumors, the immune microenvironment of intracranial tumors is unique and highly specific. The specific immune cells in the intracranial TIME mainly include microglia and astrocytes, showing heterogeneity (43). The TIME of BM is generally immunosuppressive compared to the primary foci of lung. Although there is no research on the impact of SRT on the immune microenvironment of lung cancer BM, a case report (44) showed an extracranial abscopal effect after BM stereotactic radiotherapy as second-line treatment with atezolizumab in a patient with lung adenocarcinoma. These need to be explored in large, randomized studies.

### Effects of SRT on the immune microenvironment targeting the dominant population

The immune microenvironment varies in different tumors, pathological types, and stages of tumor progression. The effect of SRT on the TIME is also different. Thus, more targeted studies are needed to identify the dominant population and to implement individualized treatment to maximize the clinical benefits.

### Study of the immune microenvironment of SRT to solve clinical problems in SRT patients

All types of studies must ultimately serve the clinic. SRT studies on the immune microenvironment aim to solve the clinical problems of SRT patients, improve clinical outcomes to a maximum extent, and contribute to the eventual implementation of survival benefits.

## Author contributions

Concept and design: HZ. Drafting and revision of the manuscript: YX. Obtained funding: HZ. All authors contributed to the article and approved the submitted version.

## Funding

This work was jointly supported by the National Key Research and Development Program of China for the development of the proton beam transmission system and proton therapy nozzle (No. 2019YFF01014403).

## Conflict of interest

The authors declare that the research was conducted in the absence of any commercial or financial relationships that could be construed as a potential conflict of interest.

## Publisher’s note

All claims expressed in this article are solely those of the authors and do not necessarily represent those of their affiliated organizations, or those of the publisher, the editors and the reviewers. Any product that may be evaluated in this article, or claim that may be made by its manufacturer, is not guaranteed or endorsed by the publisher.
